# Congenital Heart Defects and Mental Health: Stress, Psychological Treatment Use, and COVID-19-Related Burden in Young Patients—Lessons from the P-BAHn Study

**DOI:** 10.3390/jcm15114342

**Published:** 2026-06-04

**Authors:** Paul C. Helm, Jule Josephine Oster, Claudia Niessner, Ann-Kathrin Napp, Franziska Reiß, Anne Kaman, Ulrike Ravens-Sieberer, Julia Remmele, Daniel T. Marggrander, Kim Sarah Fritz, Anna-Lena Ehmann, Jannos Siaplaouras, Constanze Pfitzer, Christian Apitz

**Affiliations:** 1 National Register for Congenital Heart Defects, Augustenburger Platz 1, 13353 Berlin, Germany; 2 Deutsches Herzzentrum der Charité, Department of Congenital Heart Disease—Pediatric Cardiology, 13353 Berlin, Germany; 3 Charité—Universitätsmedizin Berlin, Corporate Member of Freie Universität Berlin and Humboldt-Universität zu Berlin, Berlin, Germany; 4 Institute of Sports and Sports Science (IfSS), Karlsruhe Institute of Technology, 76131 Karlsruhe, Germany; 5 Department of Child and Adolescent Psychiatry, Psychotherapy, and Psychosomatics, Research Section “Child Public Health”, University Medical Center Hamburg-Eppendorf, 20251 Hamburg, Germany; 6 Competence Network for Congenital Heart Defects, 13353 Berlin, Germany; 7 Department of Congenital Heart Disease and Pediatric Cardiology, German Heart Center, TUM University Hospital, 80636 Munich, Germany; 8 Technical University of Munich, Chair of Preventive Pediatrics, 80992 Munich, Germany; 9 University of Applied Sciences Fulda, Health Sciences, 36037 Fulda, Germany; 10 Division of Paediatric Cardiology, University Children’s Hospital Ulm, 89075 Ulm, Germany

**Keywords:** congenital heart defect (CHD), mental health, psychotherapy, treatment, COVID-19, young patients

## Abstract

**Background:** Congenital heart defects (CHD) are prevalent, affecting 1% of live births globally. Despite improved survival rates, adults with CHD face increased risks of psychological distress and neurocognitive deficits. The P-BAHn study (P-BAHn = “Psyche Bei Angeborenen Herzfehlern”, Psyche for congenital heart defects) evaluates the mental health status and psychosocial challenges of German children and adolescents with CHD, focusing on retrospectively assessed COVID-19-related burden and patient-/parent-rated experiences with psychological, psychotherapeutic, or psychiatric treatment (PST). **Methods:** A cross-sectional, online-based survey was conducted using the National Register for Congenital Heart Defects (NRCHD). The final dataset comprised 1567 respondent-level records from 1310 families, including 992 parent reports and 575 self-reports from children/adolescents aged 6 to <18 years. The survey assessed mental health, emotional well-being, psychosocial status, demographics, medical history, and psychological treatment. Data were analyzed descriptively using chi-square tests and *t*-tests for exploratory unadjusted group comparisons. In addition, exploratory multivariable logistic regression analyses were performed for selected key outcomes. **Results:** School-related stress was common in young CHD patients (45.3%) and was associated with older age and female sex (51.5% female vs. 35.6% male) in adjusted analyses. Overall, 17.0% of patients reported having a mental illness, most commonly anxiety (6.8%), eating disorders (5.6%), and depression (4.7%); neither sex nor CHD severity was significantly associated with self-reported mental illness in adjusted analyses. Less good/poor self-rated health was associated with older age and complex CHD in both patient and parent reports. Retrospectively assessed pandemic-related changes were perceived as quite or extremely stressful by 23.9% of patients. High COVID-19-related burden was associated with female sex, whereas CHD severity was not significant after adjustment. Among patients with previous or current PST, patient- and parent-rated treatment benefit varied by patient sex and CHD complexity. Previous/current PST was reported by 25.9% of patients and 23.8% of parents and was associated with older age in both respondent groups and with complex CHD in parent reports. Among patients with previous/current PST, 56.4% reported high perceived support. **Conclusions:** The P-BAHn study highlights the need for targeted psychosocial support for children and adolescents with CHD, including female patients, those with complex conditions, and patients reporting school- or crisis-related burden. Retrospectively reported pandemic-related burden underscores the importance of integrating crisis-sensitive strategies into psychosocial care frameworks. Longitudinal studies are essential to understand mental health trajectories and to evaluate the sustained patient- and parent-perceived benefit as well as clinical effectiveness of PST use. Enhancing support services and refining intervention models will improve the well-being and quality of life for young CHD patients.

## 1. Introduction

Congenital heart defects (CHD) are the most common congenital disorder, affecting approximately 1% of live births worldwide [1]. Advances in medical care have significantly improved survival rates, with around 90% of CHD patients now reaching adulthood [2,3]. Despite these improvements, adults with CHD (ACHD) face increased risks of psychological distress, neurocognitive deficits, and social difficulties [4,5,6]. Numerous studies have shown that ACHD patients exhibit a higher prevalence of mental health disorders compared to their healthy peers [7,8,9,10], and as they transition into adulthood, addressing psychosocial aspects becomes as crucial as medical care [11]. Nevertheless, despite empirical evidence of an increased risk of mental illness and the expressed interest in psychological treatment among those affected, research on treating psychological stress in CHD remains insufficient [12,13].

Miles et al. [14] reported that over one-third of children (<18 years) with CHD in Denmark were diagnosed or treated for a mental health condition, with a cumulative incidence of 35.1% by age 18—a rate significantly higher than that observed in the general population and sibling cohorts. Similarly, Gonzalez et al. [15] analyzed data from a large U.S. tertiary care hospital and found that 18.2% of children with CHD had an anxiety or depression diagnosis or received medication, compared to 5.2% of those without CHD. Their study concluded that youth with CHD, regardless of severity, have significantly higher odds of anxiety, depression, and ADHD than those without CHD [15]. Consequently, continuous, lifelong holistic care for CHD patients is undisputed [1]. Beyond medical management, such care should also include preventive aspects related to physical activity, nutrition, and mental well-being. Previous nationwide studies from our research group have shown reduced physical activity and specific nutritional challenges in children with CHD, particularly in those with more complex defects [16,17]. However, research on the mental health status of children and adolescents with CHD remains limited, particularly in Germany.

Global crises have increased in recent years, significantly affecting children’s and adolescents’ health, with some being more vulnerable than others. The German population-based longitudinal COPSY study (COVID-19 and PSYchological Health) found that girls, single-parent children, and those with highly burdened parents had higher multiple health complaints (MHC) during and after the COVID-19 pandemic, while social support and a positive family climate helped reduce MHC [18]. Further COPSY analyses also showed impairments in health-related quality of life (HrQoL), mental health problems, and depressive and anxiety symptoms in the context of ongoing global crises [19]. These findings emphasize the need for targeted health interventions to protect young people in future crises. Globally, the pandemic imposed an emotional burden that has negatively impacted psychological functioning and mental health [20,21,22]. Moreover, the COVID-19 pandemic likely compounded the existing stressors associated with CHD, further impairing the mental well-being of the affected young patients [23,24]. Given the chronic nature of CHD and the extra strain from pandemic-related stressors, it is critical to comprehensively assess the mental health status of children and adolescents with CHD and evaluate the adequacy of current psychological support services. If feelings are not properly perceived or effective coping strategies are lacking, significant difficulties and psychological stress can arise [25,26]. Accordingly, it is necessary to take into account possible long-term consequences, coping, and processing of the COVID-19 pandemic when analyzing the current mental health status of young CHD patients.

The P-BAHn study (“Psyche Bei Angeborenen Herzfehlern”) aims to provide a large nationwide registry-based assessment of psychological well-being, psychosocial burden, and mental health-related care use among children and adolescents with CHD in Germany. In this initial descriptive report, we focus on school-related stress, crisis- and COVID-19-related burden, self-rated health, life satisfaction, reported mental health problems, and previous or current PST. We further describe patient- and parent-rated perceptions of support, helpfulness, and long-term improvement associated with PST and explore whether key outcomes vary by patient sex and CHD severity. These analyses are intended to characterize mental health-related burden and care experiences in young patients with CHD, while avoiding causal interpretation or formal hypothesis testing. Study findings may help inform targeted psychosocial support services for this population.

## 2. Methods

### 2.1. Study Design

This cross-sectional, online-based survey targeted children and adolescents with CHD. Data were collected in the second quarter of 2024. Participants were recruited through the National Register for Congenital Heart Defects (NRCHD) [27]. Invitations were sent via email or postal mail to 10,744 eligible individuals aged 6 to <18 years. Inclusion criteria were a CHD diagnosis, age between 6 and <18 years, online consent, sufficient German reading and comprehension skills, and internet access during the study period. Within the NRCHD, minor children are registered by their parents or legal guardians, who provide consent for the child’s registration and indicate whether they themselves and/or the child may generally be contacted regarding future studies. Before starting the online survey, participants received study information and data protection information. Consent to participate in the present study was provided electronically by proceeding to and completing the online questionnaire. Accordingly, the analytical dataset consisted of respondent-level records rather than exclusively independent patient-level observations. In families in which both a parent and the affected child/adolescent participated, two respondent perspectives were available for the same family.

The online survey was pseudonymized using a nine-digit family code, which enabled the identification of possible parent–child pairs and linkage with medical data from the NRCHD database via a trusted third party. The trusted third party managed the linkage between survey data and registry-based medical information so that the research team analyzed only pseudonymized datasets and did not directly access identifying information during analysis. After entering the family code, respondents indicated whether the questionnaire was completed by the patient, a parent, or another adult caregiver. Parents and other adult respondents completed the parent-report version, whereas patients were assigned to one of four age-adapted self-report versions (6–7, 8–10, 11–13, and 14–17 years). The questionnaire covered demographic characteristics, mental health, emotional and psychosocial well-being, family and school context, crisis-related burden, COVID-19-related experiences, physical fitness, HRQoL, CHD-related functional impact, and current or previous PST. Depending on age and respondent type, completion time ranged from approximately 10 to 30 min. To reduce respondent burden and potential survey fatigue, age-adapted questionnaire versions and filter questions were used so that participants were only presented with items relevant to their age group and respondent status. Core medical information, including CHD diagnosis and severity, was obtained from the NRCHD database. If both a parent and child from the same family participated, their responses could be linked pseudonymously via the family code. However, the present analyses focused on overall group-level patterns in parent reports and patient self-reports rather than on matched parent–child dyads. Parent and patient reports were therefore treated as complementary respondent perspectives. Combined descriptive information was used only to characterize the overall respondent dataset and should not be interpreted as independent patient-level inference.

### 2.2. Measuring Instruments

The online questionnaire was developed based on the research group’s clinical and research expertise and in alignment with established instruments and large-scale survey approaches. In particular, items were adapted from the COPSY study (autumn 2023) [19] where appropriate for children and adolescents with CHD and complemented by study-specific questions. In addition, validated instruments, including the Strength and Difficulties Questionnaire (SDQ) [28], KIDSCREEN-27 [29], and the short version of the Congenital Heart Disease Specific Inventory (CHDSI) [30] were incorporated.

Separate questionnaire versions were used depending on respondent type and age. The child/adolescent self-report versions comprised 27 items for ages 6–7 years, 54 items for ages 8–10 years, and 184 items for ages 11–13 and 14–17 years, with only minor age-specific adaptations in wording and response options in the oldest group. The parent-report version comprised 271 items. Not all respondents were presented with all items, as filter questions were used to route participants to relevant sections. Response formats included single-choice items, multiple-choice items, matrix questions, scale-based ratings, and open-ended text responses.

The questionnaire covered demographic and contextual variables (e.g., age, gender, school situation), mental health and well-being, family and school context, perceived burden related to current crises, life satisfaction, diagnosed mental health problems, PST, and possible consequences of the COVID-19 pandemic, including perceived physical, psychological, and social changes after infection. Although validated instruments such as the SDQ, KIDSCREEN-27, and CHDSI were included in the broader P-BAHn questionnaire, the present manuscript focuses on selected items and item groups addressing general mental well-being, school- and crisis-related burden, COVID-19-related experiences, PST use, and patient-/parent-rated treatment benefit. Psychometric scale-score analyses of SDQ, KIDSCREEN-27, and CHDSI were not the primary objective of this initial descriptive report and will be addressed separately in analyses specifically designed to examine validated scale scores and subscale patterns.

For PST, patients were asked whether they had ever received or were currently receiving psychological, psychotherapeutic, or psychiatric treatment, whereas parents were asked the same question with regard to their child with CHD. Perceived support during PST, perceived helpfulness of PST, and perceived long-term improvement after PST were rated retrospectively on a scale from 1 (very poor) to 6 (very good), with ratings categorized as low (1–2), moderate (3–4), and high (5–6). These ratings reflect subjective patient- or parent-reported benefit and should not be interpreted as prospective evidence of treatment efficacy.

### 2.3. National Register for Congenital Heart Defects

As of March 2025, the NRCHD manages medical data for approximately 60,000 CHD patients [31], making it Europe’s largest database for CHD [32]. It plays a key role in clinical research, facilitating studies on various aspects of CHD. Participation is voluntary and requires patients’ general consent, allowing treating physicians to transmit medical information. This consent applies to both current and future research but can be revoked at any time. The NRCHD’s multicenter research approach enables large nationwide registry-based data collection and analysis on CHD and mental health. Previous analyses have demonstrated that the NRCHD provides broad clinical coverage of the German CHD population, making it particularly suitable for nationwide registry-based research [32]. Nevertheless, participation in the present survey was voluntary, and possible response bias must be considered.

### 2.4. Statistical Analyses

Statistical analyses were carried out using SPSS (Version 29.0). Data were analyzed primarily using descriptive statistical methods. All group comparisons were exploratory and unadjusted. Exploratory group comparisons by patient sex and CHD severity were conducted using chi-square tests for categorical variables, while pairwise *t*-tests were used for continuous or approximately continuous variables. Ordinal response variables that were collapsed into broader categories for analysis were treated as categorical variables and analyzed using chi-square tests. Given the exploratory nature of the analyses and the sample size, the use of *t*-tests was considered appropriate despite possible moderate deviations from normality. No correction for multiple testing was applied, as the analyses were exploratory and intended to identify potentially relevant group differences without increasing the risk of overlooking meaningful associations. To assess whether selected exploratory associations were robust to basic adjustment, additional multivariable logistic regression analyses were performed for key outcomes. These outcomes were selected a priori based on clinical relevance and included school-related stress, less good/poor self-rated health, high COVID-19-related burden, any self-reported mental illness, previous/current PST use, and moderate/high perceived long-term improvement after PST. Models were fitted separately for patient self-reports and parent reports where applicable, in order to avoid treating parent and child reports from the same family as independent observations. Predictor variables included age, sex, CHD severity, and syndromic/genetic condition where model convergence allowed. Simple CHD, male sex, and absence of syndromic/genetic condition served as reference categories. Results are presented as odds ratios (ORs) with 95% confidence intervals. All multivariable analyses were exploratory and were not corrected for multiple testing.

CHD cases were assigned a principal diagnosis based on the International Pediatric and Congenital Cardiac Code (IPCCC) [33] and categorized as simple, moderate, or complex CHD according to the severity classification of Warnes et al. [34]. Gender differences were based on the biological sex recorded in medical documents (e.g., doctors’ letters).

### 2.5. Ethical Statement

Ethical approval for this study (EA2/197/22) was given by the Ethics Committee of Charité Berlin. Participation was voluntary and based on the NRCHD consent procedures, including parental registration of minor children and permission for study contact, as well as study-specific electronic consent after provision of study and data protection information at the beginning of the online survey.

## 3. Results

**Figure 1:**
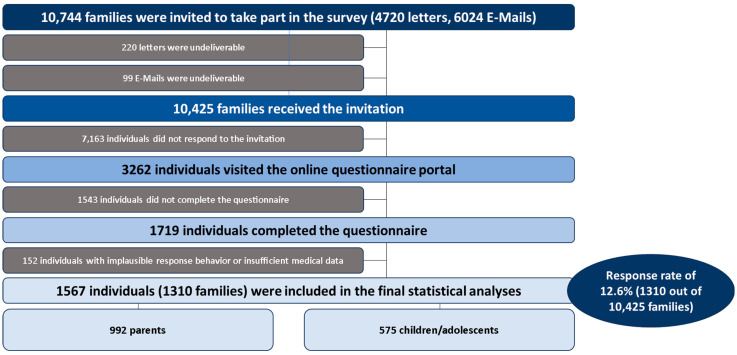
Sample recruitment.

Of the 10,744 families invited, 10,425 successfully received the invitation. A total of 3262 respondents accessed the questionnaire portal, but 1543 did not complete the survey, leaving 1719 completed responses. After excluding 152 responses because of implausible response behavior or insufficient medical data, the final analysis included 1567 respondent-level records from 1310 families, comprising 992 parent reports and 575 self-reports from children/adolescents with CHD. Further details on sample recruitment are provided in Figure 1.

A sociodemographic and clinical overview of the total sample is shown in Table 1. Across the 1567 respondent-level records, 51.4% referred to female patients, with a mean patient age of 13.0 ± 3.5 years. With regard to CHD severity, 31.2% of patients had simple CHD, 31.7% moderate CHD, and 37.1% complex CHD. In addition, 104 of the 1567 represented patients (6.6%) had a documented syndromic or genetic condition. These conditions were more frequently represented in the parent-report group than in the self-report group (88/992 [8.9%] vs. 16/575 [2.8%]).

Among the included responses, 257 families contributed both a parent report and a child/adolescent self-report. However, the present analyses focused on group-level comparisons between all parent reports and all patient self-reports rather than on matched parent–child dyads.

To contextualize the composition of the final analysis dataset, basic characteristics of all invited patients were compared descriptively with those of the included respondent-level records. Among all 10,744 invited patients, 49.1% were female and 50.9% were male, with a mean age of 13.21 ± 3.46 years. The final analysis dataset included 51.4% female and 48.6% male patient records, with a mean age of 13.0 ± 3.5 years.

With regard to CHD severity, the invited cohort comprised 36.3% simple, 26.4% moderate, 23.0% complex, and 14.2% non-classified cases. After excluding non-classified cases from the invited cohort, the distribution was 42.4% simple, 30.8% moderate, and 26.8% complex CHD. In the final analysis dataset, 31.2% of patient records were classified as simple CHD, 31.7% as moderate CHD, and 37.1% as complex CHD.

### 3.1. Mental Health and Wellbeing

Of the 575 self-responding young CHD patients, 528 were in school at the time of the survey. A total of 503 patients were aged 11 years or older (mean age 15.3 ± 1.8 years) and were therefore asked about their current feelings toward school, studying, or working. 45.3% found it somewhat or very stressful, 35.0% moderately stressful, and 19.7% not very or not at all stressful. Both patient sex and CHD severity were significantly associated with responses. Female patients were more likely to report school and learning as somewhat or very stressful (51.5% vs. 35.6% for males, *p* = 0.0007). Patients with simple CHD were the most likely to find school somewhat or very stressful (51.1%), followed by those with moderate (40.9%) and complex CHD (40.8%) (*p* = 0.032). Exploratory adjusted analyses of school-related stress and other key outcomes are presented together in Section 3.5.

Patients (N = 503) rated their overall physical fitness on a scale from 1 (very unfit) to 10 (very fit), with a mean score of 6.4 ± 2.2. In exploratory pairwise comparisons by CHD severity, patients with moderate CHD reported slightly higher fitness scores than those with complex CHD (6.7 ± 2.2 vs. 6.2 ± 2.2; *p* = 0.044), while no significant differences were observed between patients with simple CHD (6.4 ± 2.1) and those with moderate or complex CHD. Male patients reported significantly higher fitness scores than female patients (6.7 ± 2.1 vs. 6.2 ± 2.2; *p* = 0.019).

Of the 503 patients aged 11 years or older, 2.8% described the family mood as bad or very bad, 24.3% rated it as average, and 73.0% considered it good or very good. Neither patient sex nor CHD severity was significantly associated with these perceptions. Among the 992 parents surveyed, the distribution of ratings of the current family mood was descriptively similar: 3.4% described the family mood as bad or very bad, 24.5% rated it as average, and 72.1% considered it good or very good. Neither patient sex nor CHD severity was significantly associated with parents’ perceptions.

Out of the 503 CHD patients, 12.3% rated their overall health as less good to poor, 41.4% considered it good, and 46.3% described it as very good or excellent. Patient sex and CHD severity were significantly associated with response behavior (*p* = 0.002 and *p* = 0.045), with female patients tending to rate their health lower than male patients. In fact, 56.2% of male patients described their health as very good or excellent compared to 40.1% of female patients, while a higher proportion of female patients assessed their health as good (45.6% vs. 34.5%) or less good/poor (14.2% vs. 9.3%). Patients with complex CHD were the least likely to report very good or excellent health (41.4%), compared with those with simple (45.2%) or moderate CHD (53.8%). Among the 992 parents surveyed on how their child would describe their own health, the distribution of ratings was descriptively similar: 10.6% rated it as less good to poor, 47.8% as good, and 41.6% as very good or excellent. CHD severity was significantly associated with parents’ responses (*p* = 0.0003), with parents of patients with complex CHD being the least likely to report very good or excellent health (34.9%), compared with those with simple (47.3%) or moderate CHD (45.1%). The corresponding proportions for good overall health were 43.8% (simple CHD), 48.1% (moderate CHD), and 50.1% (complex CHD), and for less good/poor health, 8.8% (simple CHD), 6.8% (moderate CHD), and 14.9% (complex CHD), respectively. Corresponding multivariable models for patient- and parent-rated health are summarized in Section 3.5.

Among 503 patients, 39.0% reported that changes related to the coronavirus pandemic were not at all or hardly stressful, 37.2% found them somewhat stressful, and 23.9% considered them quite or extremely stressful. Both patient sex (*p* = 0.0004) and CHD severity (*p* = 0.008) were significantly associated with perceived pandemic-related burden, with females reporting higher overall burden than males (29.8% high burden vs. 14.4% of males). Similarly, patients with complex CHD felt the most distressed (27.0% reported high distress vs. 26.0% simple CHD and 16.7% moderate CHD). An additional exploratory analysis by age group (11–13, 14–15, and 16–17 years) showed no significant overall group difference in the distribution of perceived pandemic-related burden (chi-square *p* = 0.125), but a significant linear trend indicated that older patients tended to report greater burden (linear-by-linear association *p* = 0.014). Adjusted analyses of high COVID-related burden are presented in Section 3.5.

The 992 parents were also asked to rate how stressful pandemic-related changes had been for them personally. 29.5% reported that changes were not at all or hardly stressful, 34.4% found them somewhat stressful, and 36.1% considered them quite or extremely stressful. Neither the child’s sex nor CHD severity was significantly associated with parents’ perceptions.

Among the 575 patients, 6.6% reported that someone in their family or social circle had died from COVID-19; a similar proportion was reported in the parent reports (6.4%). Overall, 72.5% of patients and 74.3% of parents reported that the child had experienced a COVID-19 infection. The 417 patients and 737 parents in these subgroups were subsequently asked to assess the child’s condition in four specific domains by comparing the period before and after the infection.

In the parent reports, most respondents observed no change in their child’s physical resilience, psychological state, social environment, or general health following COVID-19 infection. However, a notable minority reported worsening, particularly in psychological state (18.9%) and general health (17.9%). Physical resilience was most often rated as unchanged (86.3%), although 9.8% of parents reported a decline. Parents of female patients were significantly more likely than parents of male patients to report worsening physical resilience in their child (*p* = 0.018). In the patients’ self-reports, a higher proportion described declines in physical resilience (16.5%) and general health (19.9%) than in the parent reports. A significant sex difference was observed for physical resilience (*p* = 0.0004), with female patients reporting deterioration more often than male patients. Psychological state also showed notable deterioration, particularly among patients with simple CHD (27.1%), with significant differences across CHD severity groups (*p* = 0.031). Although most patients reported no change in their social environment, a considerable proportion (19.4%) described improvement. Further details are provided in Table 2.

Patients rated the burden associated with current crises, including wars, economic instability, energy concerns, climate change, and terrorism, while parents assessed the burden experienced by their child. Among the 503 CHD patients, 55.1% found these crises not at all or hardly stressful, 31.8% somewhat stressful, and 13.1% quite or extremely stressful. Sex differences were significant (*p* = 0.007), with female patients perceiving crises as more challenging than males. Specifically, 49.5% of females found them not at all or hardly stressful (vs. 63.9% of males), 35.6% somewhat stressful (vs. 25.8%), 14.9% quite or extremely stressful (vs. 10.3%). Parents also evaluated the overall crisis burden for their children. Among 992 parents, 71.6% considered the crises not at all or hardly stressful, 23.2% somewhat stressful, and 5.2% quite or extremely stressful. Neither patient sex nor CHD severity was significantly associated with parental assessments.

Patients (N = 503) rated their overall life satisfaction on a scale from 0 (worst) to 10 (best), with a mean score of 8.0 ± 1.8. Male patients reported significantly higher life satisfaction than female patients (8.3 ± 1.7 vs. 7.8 ± 1.8; *p* = 0.002). In exploratory pairwise comparisons by CHD severity, patients with simple CHD reported significantly lower life satisfaction than those with moderate CHD (7.8 ± 1.8 vs. 8.2 ± 1.8; *p* = 0.03), whereas patients with complex CHD (8.1 ± 1.8) did not differ significantly from the other CHD severity groups.

Of all 575 patients, 17.0% reported having a mental illness. Specifically, 39 (6.8%) reported anxiety symptoms, an anxiety disorder, or panic attacks; 32 (5.6%) reported an eating disorder; 27 (4.7%) reported depressive symptoms or depression; 16 (2.8%) reported concentration problems (e.g., ADHD); and 29 (5.0%) reported another mental health condition. Adjusted analyses of self-reported mental illness are presented in Section 3.5.

Among the 992 parents surveyed, 11.7% reported having a mental illness themselves. Specifically, 56 (5.6%) reported depressive symptoms or depression, 25 (2.5%) reported anxiety symptoms, an anxiety disorder, or panic attacks, 16 (1.6%) reported stress symptoms, trauma, or PTSD, 14 (1.4%) reported exhaustion or burnout, and 21 (2.1%) reported another mental health condition.

### 3.2. Psychological, Psychotherapeutic, or Psychiatric Treatment

**Figure 2:**
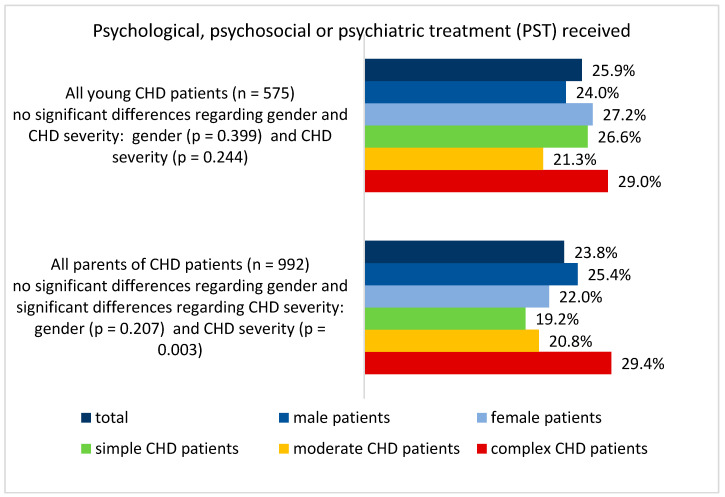
Reported rates of psychological, psychotherapeutic, or psychiatric treatment (PST) among CHD patients.

Overall, reported PST rates did not differ significantly by patient sex, but CHD severity was associated with reported PST rates in the parent reports. Specifically, parents of children with complex CHD were significantly more likely to report that their child had received PST than parents of children with simple or moderate CHD. In contrast, CHD severity was not significantly associated with self-reported PST rates among young CHD patients. Reported PST rates were 23.8% in the parent reports and 25.9% in the patient self-reports. The highest reported PST rates were observed for patients with complex CHD, ranging from 29.0% in patient self-reports to 29.4% in parent reports. Further details are provided in Figure 2. Adjusted analyses of previous/current PST use are presented in Section 3.5.

### 3.3. Further Questions on Psychological, Psychotherapeutic, or Psychiatric Treatment

In total, 149 of the 575 surveyed patients (25.9%) reported current or previous PST, and 236 of the 992 surveyed parents (23.8%) reported current or previous PST for their child with CHD. If PST was reported, further questions were asked. Reported reasons for PST are summarized in Table 3. Overall, school-related problems, mental illness, family- or peer-related problems, and other reasons were among the most frequently reported indications in both parent and patient reports. In the parent reports, attributing PST to the CHD itself differed significantly by CHD severity (*p* = 0.0004) and was reported most often for children with complex CHD. In the patient reports, attributing PST to the CHD itself also differed significantly by sex (*p* = 0.005) and CHD severity (*p* = 0.004), with male patients and those with complex CHD reporting this reason more often.

Among the 149 CHD patients who were currently receiving or had previously received PST, the majority (80.5%) reported that the treatment had been provided by a psychologist or psychotherapist. In comparison, 12.1% reported that the treatment had been provided by a physician, 5.4% by another person, and 2.0% were unsure. A similar distribution was observed in the parent reports: among the 236 parents who reported PST for their child with CHD, 79.2% stated that the treatment had been provided by a psychologist or psychotherapist, 16.5% by a physician, and 4.2% by another person.

Among the 148 patients aged eight years or older, recommendations for PST were reported to come from various sources; most commonly family or friends (31.1%), a physician (29.1%), or the patients themselves (23.6%), 8.8% named another source, and 7.4% were unsure who had recommended PST. Among the parents who reported PST for their child with CHD, treatment initiation was predominantly described as parent-driven: 64.4% stated that the decision had been made on their own initiative, 25.8% reported a physician’s recommendation, 8.1% named another source, and 1.7% cited family or friends.

In the parent reports, 89.4% stated that their child’s PST was fully covered by health insurance, while 5.5% reported partial coverage and 5.1% reported full out-of-pocket payment. Overall, 45.3% stated that their child had attended between 1 and 10 appointments, 17.8% reported 11 to 20 sessions, and 36.9% reported more than 20 appointments.

### 3.4. Patient- and Parent-Rated Benefit of Psychological, Psychotherapeutic, or Psychiatric Treatment

Overall, many patients and parents retrospectively rated PST as supportive and helpful, although patient- and parent-rated benefit varied according to patient sex and CHD severity. In the parent reports, 55.9% rated the level of support their child received during PST as high, 36.4% as moderate, and 7.6% as low. Parents of male patients were significantly less likely than parents of female patients to report a high level of support (*p* = 0.007). Regarding perceived helpfulness, 47.5% of parents rated PST as highly helpful, 36.4% as moderately helpful, and 16.1% as low in helpfulness. For perceived long-term improvement, 40.7% of parents reported high improvement, 37.7% moderate improvement, and 21.6% low improvement. Parents of female patients reported significantly greater long-term improvement than parents of male patients, with high perceived long-term improvement reported for 48.5% of female patients compared with 34.8% of male patients (*p* = 0.005). CHD severity was also associated with perceived long-term improvement: parents of patients with simple CHD reported high perceived improvement most frequently (52.0%), followed by parents of patients with moderate CHD (44.3%) and complex CHD (33.6%) (*p* = 0.037). Patient reports showed a distribution similar to that observed in the parent reports (see Table 4). Adjusted analyses of perceived long-term improvement after PST are presented in Section 3.5.

### 3.5. Exploratory Multivariable Logistic Regression Analyses of Key Outcomes

Exploratory multivariable logistic regression analyses were performed for selected key outcomes to assess whether the main descriptive associations were robust to basic adjustment for age, sex, CHD severity, and syndromic/genetic condition where model convergence allowed. Models were fitted separately for patient self-reports and parent reports where applicable. Full results are shown in Table 5.

In patient self-reports, older age and female sex were associated with higher odds of school-related stress, whereas CHD severity was not significantly associated with school-related stress after adjustment. Less good/poor self-rated health was associated with older age and complex CHD in patient self-reports, and a corresponding parent-report model showed the same pattern, although with a smaller effect size for complex CHD. High COVID-19-related burden was associated with female sex, while CHD severity was not significantly associated with this outcome after adjustment. The model for any self-reported mental illness did not identify significant associations with sex or CHD severity; age showed a non-significant tendency toward higher odds.

For previous/current PST use, older age was associated with higher odds in both patient self-reports and parent reports. In parent reports, complex CHD was additionally associated with higher odds of reported PST use, whereas this association was not significant in patient self-reports. Among respondents with previous/current PST, no individual predictor was significantly associated with moderate/high perceived long-term improvement in patient self-reports. In parent reports, female patient sex was associated with higher odds of moderate/high perceived long-term improvement, while complex CHD showed a non-significant tendency toward lower odds.

## 4. Discussion

The P-BAHn study provides important insights into mental health status, psychosocial challenges, crisis-related burden, use of PST, and patient- and parent-rated benefit of PST among German children and adolescents with CHD. Our findings show that mental health problems and psychosocial burden are common in this population, with both CHD severity and patient sex being associated with perceived stress, overall health, and treatment-related outcomes.

Because this was a cross-sectional, exploratory survey based on self- and parent-reported data, all associations should be interpreted as descriptive and hypothesis-generating. The multivariable models were intended to examine the robustness of selected associations to basic adjustment, not to establish causality.

The comparison between the invited cohort and the final analysis dataset suggests that age and sex distributions were broadly similar. However, patients with complex CHD were more strongly represented in the final analysis dataset, whereas patients with simple CHD were less strongly represented. This pattern should be considered when interpreting the descriptive prevalence estimates and limits unqualified claims of representativeness for the overall population of invited children and adolescents with CHD.

### 4.1. Mental Health and Wellbeing

The findings indicate several areas of psychosocial burden among young CHD patients, particularly school-related stress, crisis-related burden, reduced self-rated health and fitness, lower life satisfaction in female patients, and self-reported mental health conditions. School-related stress affected 45.3% of self-reporting patients aged 11 years or older and differed by patient sex and CHD severity in unadjusted analyses. Female patients reported more stress than males (51.5% vs. 35.6%), and unexpectedly, patients with simple CHD reported the highest proportion of school-related stress. However, this severity-related association was attenuated in multivariable logistic regression and was no longer statistically significant after adjustment. In contrast, older age and female sex remained associated with higher odds of school-related stress. This suggests that the initially observed severity-related pattern may partly reflect other unmeasured factors rather than an independent effect of anatomical CHD severity. Nevertheless, the descriptive finding remains clinically relevant because it indicates that school-related stress is not confined to patients with complex CHD. Patients with anatomically less severe CHD may also experience substantial psychosocial burden, potentially related to lower illness visibility, fewer formal accommodations, less anticipatory psychosocial guidance, or higher school-related expectations. Chiang et al. [35] found that adolescents with mild CHD transition from feeling limited to mastering their condition through self-awareness and coping strategies. These insights, together with the present findings, highlight the need for early psychosocial support and tailored supportive interventions.

Self-rated fitness averaged 6.4/10, with males rating themselves higher than females (6.7 vs. 6.2, *p* = 0.019). CHD severity was also associated with perception, with patients with moderate CHD rating their fitness slightly higher than those with complex CHD. This pattern is in line with population-based findings from Germany: data from the MoMo study show pronounced sex differences in physical activity participation, particularly in sports club activity, with boys generally being more active than girls; MoMo-based analyses further highlight the relevance of physical self-concept for adolescents’ activity behavior [36,37].

Regarding health perception, 12.3% rated their health as less good to poor, with females and patients with complex CHD reporting the most negative views. Multivariable analyses supported the relevance of CHD severity for self- and parent-rated health. After adjustment, complex CHD remained associated with higher odds of less good/poor health in both patient self-reports and parent reports. This consistency across respondent perspectives suggests that the less favorable health perception among patients with complex CHD is not explained solely by age or sex differences in the present sample. In an informal contextual comparison with data from the German KiGGS study, this proportion appears higher: in KiGGS Wave 2, only 4.3% of 3- to 17-year-olds were rated as having fair/bad/very bad general health overall, and even among 14- to 17-year-olds, the proportion was 6.8% in girls and 4.8% in boys. However, this comparison is not age- or sex-standardized and should be interpreted cautiously because the present study and KiGGS differ in age range, respondent type, item wording, assessment period, and clinical composition. Although direct comparisons are limited, the contrast suggests that adolescents and young adults with CHD—especially females and those with complex CHD—perceive their health less favorably than peers from the general population [38]. Taken together, these findings underscore the impact of patient sex and CHD severity on fitness and health perceptions and suggest that disease burden adds to the sex differences already observed in the general population [36,37,38].

Among patients aged 11 years or older, 73.0% rated family mood as good or very good. Parent reports showed descriptively similar distributions, suggesting that family climate may be an important contextual factor for mental well-being. However, female patients more often reported a high burden related to current crises than male patients (14.9% vs. 10.3%). Jackson et al. [39] reported that CHD families cope in diverse ways, influenced by psychosocial resources and family cohesion rather than disease severity. Strong parental support fosters psychological well-being, while resource-limited families face greater distress. The present study’s high family mood provides a foundation for strengthening resilience and adaptive coping strategies.

Retrospectively assessed pandemic-related burden differed between subgroups, with female patients and those with complex CHD reporting greater distress, whereas parents’ perceptions of pandemic-related burden were not significantly associated with the child’s sex or CHD severity. An additional exploratory analysis by age group (11–13, 14–15, and 16–17 years) showed no significant overall group difference in perceived pandemic-related burden (*p* = 0.125). However, a significant linear trend was observed (*p* = 0.014), indicating that older patients tended to report greater burden related to pandemic-associated changes. In multivariable analysis, female sex remained strongly associated with high COVID-related burden, whereas CHD severity was no longer statistically significant after adjustment. The robust association with female sex is consistent with broader population-based findings showing higher psychosocial burden among girls and female adolescents during and after the pandemic. However, because COVID-19-related burden was assessed retrospectively in 2024, these findings should not be interpreted causally.

Overall, 72.5% of patients reported having had COVID-19, and 74.3% of parents reported COVID-19 infection in their child. Because these assessments were retrospective, the reported changes cannot be attributed causally to COVID-19 infection. In the parent reports, most respondents did not observe substantial changes in their child after infection, although a notable minority reported declines in psychological state (18.9%) and general health (17.9%). In the patient self-reports, deterioration was also reported, particularly for physical resilience (16.5%) and general health (19.9%), with female patients being especially affected. Similar to the findings on school-related stress, this pattern may reflect differences in baseline mental health, family context, expectations, follow-up intensity, illness visibility, or selection effects rather than a direct effect of CHD severity.

Social consequences of COVID-19 appeared heterogeneous. Although most patients reported no change in their social environment, 19.4% described improvements, which may reflect reduced social pressures or altered social demands during and after the pandemic. These findings underline the need for continued psychological support for young CHD patients, particularly for female patients and those with complex CHD.

Harvey reported high mental health burdens among parents of young children with CHD during the COVID-19 pandemic, while identifying resilience as well as emotional and informational support as important protective factors [40]. Moons et al. [41] found that adults with CHD in Belgium, Norway, and South Korea experienced considerable pandemic-related disruptions but also demonstrated resilience. In contrast, the present findings suggest that younger CHD patients may have experienced more persistent psychosocial burden in specific domains. Together with previous nationwide analyses on physical activity and nutritional status in children with CHD, the present findings contribute to a broader understanding of modifiable and psychosocial aspects of long-term care in this population.

Life satisfaction averaged 8.0/10, with males (8.3) reporting higher satisfaction than females (7.8). This sex difference is broadly consistent with findings from the German HBSC study, although the comparison is informal and not age- or sex-standardized, which also identified girls as being at greater risk of lower well-being and low life satisfaction when assessed using the Cantril Ladder. In the 2022 HBSC survey, the majority of adolescents still reported high life satisfaction, but girls and older adolescents were significantly more likely to fall below the established cut-off for low life satisfaction (≤6). Direct comparisons are limited by differences in age range, clinical status, respondent type, assessment period, and item context. Nevertheless, our findings suggest that female patients with CHD may represent a particularly vulnerable subgroup with regard to subjective well-being [42]. In exploratory comparisons, patients with moderate CHD reported slightly higher life satisfaction than those with simple CHD. This pattern may reflect that psychosocial adaptation is not determined by anatomical severity alone, but also by coping processes, family context, and subjective illness experience. Self-reported mental illness prevalence was 17.0%, with anxiety (6.8%) and eating disorders (5.6%) being the most common. In contrast to school-related stress, self-rated health, and COVID-related burden, the multivariable model for a self-reported mental illness did not identify significant associations with sex or CHD severity. This may indicate that reported mental illness in this cohort is influenced by a broader range of factors not captured in the present models. It should also be considered that the outcome was based on self-reported mental illness rather than a standardized diagnostic assessment. In informal comparison with population-based estimates from Germany, this proportion does not appear markedly elevated, although such comparisons should be interpreted cautiously because of differing assessment methods and case definitions. In KiGGS Wave 2, 16.9% of children and adolescents were classified as having mental health problems based on screening data; nationwide ambulatory claims data further suggest that documented mental disorders are common in this age group. At the same time, the distribution of disorders in our sample is noteworthy, as anxiety disorders are also among the most frequent conditions in population-based German studies, while eating disorder symptoms have been reported particularly often among girls and adolescents [43,44,45,46]. Parental self-reported mental illness prevalence was lower (11.7%), but still highlights family challenges. Taken together, these findings support the need for holistic psychosocial care concepts that address not only cardiac status, but also emotional well-being, mental health, and family burden [47].

### 4.2. Psychological, Psychotherapeutic, or Psychiatric Treatment

Reported PST rates ranged from 23.8% (parent reports) to 25.9% (patient reports). While sex differences were not statistically significant, CHD severity was associated with reported PST rates, particularly in the parent reports. Parents of children with complex CHD were significantly more likely to report PST than those with milder forms, suggesting that greater disease severity may be associated with higher perceived psychosocial burden, greater awareness of mental health needs, more frequent contact with specialized care, or a higher likelihood of referral. This finding is in line with previous literature discussed in the introduction, showing that parents of children with CHD experience elevated rates of psychological distress, anxiety, depression, and post-traumatic stress symptoms, especially in the context of critical or more severe disease courses. Reviews further indicate that a substantial proportion of these families have ongoing psychosocial care needs [48]. While CHD severity had no significant effect on PST rates in young patients—possibly due to smaller subgroup sizes—those with complex CHD consistently showed the highest PST rates across groups. This pattern likewise accords with prior studies suggesting that children and adolescents with CHD and their families are a psychosocially vulnerable population, even though associations with anatomical severity are not always uniform across studies [48,49].

Parents of patients with complex CHD reported PST rates up to 29.4%, highlighting considerable psychosocial care needs in this subgroup. Recent data comparing parents of children with univentricular hearts with parents of children with simple defects also support this interpretation, showing higher psychosocial burden and reduced quality of life in families facing particularly complex cardiac conditions [48,50].

Multivariable analyses further clarified the pattern of PST use. Older age was associated with higher odds of previous/current PST use in both patient self-reports and parent reports, which may reflect a longer cumulative opportunity to receive treatment and increasing recognition of psychosocial needs during adolescence. In parent reports, complex CHD remained associated with higher odds of reported PST use after adjustment, whereas this association was not significant in patient self-reports. This difference may reflect parental awareness of cumulative disease-related burden, greater healthcare contact among families of patients with complex CHD, or differences between proxy and self-perceived treatment needs. Interestingly, a syndromic/genetic condition was associated with lower odds of parent-reported PST use. This may reflect different care pathways, such as developmental, social-pediatric, rehabilitative, or educational support services that are not perceived or reported as psychological, psychotherapeutic, or psychiatric treatment.

### 4.3. Further Questions on Psychological, Psychotherapeutic, or Psychiatric Treatment

PST reasons among young CHD patients vary, with school difficulties and unspecified factors most commonly reported by both parents and patients. Mental illness and social issues were also frequent triggers. Notably, while 25.8% of parents linked PST to CHD, only 14.9% of patients did, with males and those with complex CHD more likely to make this connection—suggesting parents may perceive CHD as a greater psychological burden than patients do.

Psychologists or psychotherapists were the primary treatment providers, underscoring the need for specialized mental health support. The predominantly parent-driven initiation of treatment also suggests that parental awareness and navigation of care pathways may be central for access to PST in this age group. Parents typically initiated treatment independently.

Regarding health insurance coverage for PST, parents reported that most treatments were fully covered. Overall, parents most frequently reported that their child had between 1 and 10 PST appointments.

Hays et al. [51] found that ACHD patients, mostly with moderate to complex CHD, valued psychological support, future planning, coping skills, and navigating the healthcare system. They emphasized education on health expectations and stress management.

The P-BAHn study reinforces the need for targeted mental health support, especially for complex CHD patients, while also highlighting parental influence in seeking care.

### 4.4. Patient- and Parent-Rated Benefit of Psychological, Psychotherapeutic, or Psychiatric Treatment

PST was generally rated as supportive and helpful by many respondents, although these ratings represent retrospective patient- and parent-perceived benefit rather than objective treatment efficacy. Over half of parents and patients reported high perceived support. Parents of male patients were less likely to report strong support for their child, and perceived long-term improvement was rated higher for female patients, which may reflect sex differences in perceived treatment benefit, treatment needs, communication, parental perception, or unmeasured characteristics of the treatment context. CHD severity was also associated with perceived long-term improvement, with higher ratings reported more often in patients with simple or moderate CHD than in those with complex CHD. In adjusted analyses among respondents with previous/current PST, patient self-reports did not identify significant individual correlates of moderate/high perceived long-term improvement. In contrast, parent reports showed that female patient sex was associated with higher odds of moderate/high perceived long-term improvement. This is consistent with the descriptive finding that parents of female patients more often reported high long-term improvement after PST. Complex CHD showed a non-significant tendency toward lower odds of moderate/high perceived improvement, suggesting that perceived long-term benefit may be more difficult to achieve or less readily perceived in the context of more complex disease burden.

Knowles et al. [52] found that children with severe CHD felt socially isolated, while those with milder conditions often viewed CHD as a minor issue or part of the past. Both groups used coping strategies, but only those with mild CHD expressed a sense of survivorship, emphasizing the need for social participation in pediatric CHD care. Jackson et al. [53] found that young adults with CHD and executive function issues experienced more distress, partly due to ineffective coping. While coping had little impact on adolescents, young adults using maladaptive strategies reported higher distress [53]. These findings suggest that coping strategies vary by disease severity and should be considered in psychological interventions.

## 5. Limitations

Several limitations should be considered when interpreting the present findings. First, the cross-sectional design precludes causal inferences regarding associations between sex, CHD severity, mental health outcomes, PST use, patient-/parent-rated treatment benefit, and COVID-19-related burden. The analyses were exploratory; no correction for multiple testing was applied, and all descriptive group comparisons were unadjusted. Although additional multivariable logistic regression analyses were performed for selected key outcomes, these models included only a limited number of a priori selected covariates and were not intended to establish causal relationships. Residual confounding by unmeasured factors such as socioeconomic status, family context, school environment, baseline mental health, healthcare access, and prior psychosocial support cannot be excluded. Some subgroup models were also limited by sparse cell counts.

Second, the reliance on self-reported and proxy-reported data may have introduced recall bias, social desirability bias, and differences between patient and parent perceptions. This is particularly relevant for the COVID-19-related items, as pandemic-related burden and perceived changes after infection were assessed retrospectively in 2024. Information on timing of infection, infection severity, vaccination status, long-COVID symptoms, and baseline mental health was not available in sufficient detail. Therefore, perceived changes after infection cannot be attributed causally to COVID-19 infection, and infection-related, pandemic-related, developmental, family-related, school-related, and broader societal effects cannot be clearly separated.

Third, although recruitment through the NRCHD enabled a large nationwide registry-based sample, participation was voluntary and required internet access and sufficient German language skills. Thus, response bias cannot be excluded. Families with higher psychosocial burden, greater interest in mental health, higher health literacy, better internet access, or stronger connection to the NRCHD may have been more likely to participate, whereas socially disadvantaged families, migrant families, and families with limited German language proficiency may be underrepresented. In addition, the final analysis dataset was broadly similar to the invited cohort in age and sex but included a higher proportion of patients with complex CHD and a lower proportion with simple CHD. Consequently, unqualified claims of representativeness should be avoided.

Fourth, the analyses were based on parent and patient reports as complementary respondent perspectives rather than being restricted to the smaller subgroup of 257 matched parent–child dyads. This allowed broader descriptive use of the available data, but formal parent–child agreement analyses and clustering-sensitive analyses were not performed. Such dyadic agreement analyses require a separate analytical framework and will be addressed in future work focusing specifically on proxy-self concordance. The descriptive distribution of syndromic/genetic conditions also differed between parent-report and self-report groups, likely reflecting differences in the ability or opportunity to participate directly in the survey. Dyadic analyses and more detailed analyses of syndromic/genetic subgroups should therefore be addressed in future work.

Finally, ratings of PST support, helpfulness, and long-term improvement were retrospective subjective assessments among respondents who reported previous or current PST. They should not be interpreted as prospective evidence of treatment efficacy. Information on treatment type, duration, therapeutic approach, treatment indication, baseline symptom severity, and standardized pre-post outcomes was limited; therefore, conclusions about clinical effectiveness cannot be drawn.

## 6. Conclusions

The P-BAHn study underscores the need for targeted psychosocial support in children and adolescents with CHD, particularly for female patients, patients with complex CHD, and those reporting school- or crisis-related burden. Although psychological, psychotherapeutic, or psychiatric treatment was retrospectively perceived as supportive and helpful by many patients and parents, these findings reflect subjective patient- and parent-rated benefit rather than objective evidence of treatment efficacy. The considerable variation in perceived support, helpfulness, and long-term improvement points to the necessity for tailored approaches that address individual needs and circumstances. Retrospectively reported pandemic-related burden further emphasizes the importance of developing resilient psychosocial care strategies that can address both chronic disease-related stress and acute external crises.

Moving forward, longitudinal studies are essential to better understand mental health trajectories in this population and to evaluate the sustained perceived benefit and clinical effectiveness of targeted psychosocial interventions. Future studies should include repeated assessments at developmentally and clinically meaningful intervals, for example, during late childhood, early adolescence, mid-adolescence, transition to adult CHD care, and early adulthood. Annual or biennial follow-up assessments may help distinguish transient crisis-related burden from persistent mental health trajectories. Enhancing psychosocial support services, refining intervention models, and incorporating crisis-specific measures will be vital to improving the overall well-being and quality of life for young CHD patients, ultimately leading to more holistic and effective lifelong care.

## Figures and Tables

**Table 1:** Sociodemographic and clinical overview of the respondent-level dataset. Data are based on 1567 respondent-level records from 1310 families and are shown for the full respondent dataset, parent reports (n = 992), and patient self-reports (n = 575). Parent-report and self-report groups are shown descriptively; no inferential comparison between respondent groups was performed because these groups reflect different respondent types and age-dependent questionnaire pathways.

	Total N = 1567	Parents n = 992	Children n = 575
Male patients	761 (48.6%)	532 (53.6%)	229 (39.8%)
Female patients	806 (51.4%)	460 (46.4%)	346 (60.2%)
Average patient age *(mean and standard deviation)*	13.0 ± 3.5 years	12.2 ± 3.6 years	14.5 ± 2.8 years
Simple CHD patients	489 (31.2%)	260 (26.2%)	229 (39.8%)
Moderate CHD patients	497 (31.7%)	337 (34.0%)	160 (27.8%)
Complex CHD patients	581 (37.1%)	395 (39.8%)	186 (32.4%)
At least one congenital cardiac comorbidity is present	1060 (67.6%)	702 (70.8%)	358 (62.3%)
At least one acquired cardiac comorbidity is present	754 (48.1%)	527 (53.1%)	227 (39.5%)
At least one extracardiac diagnosis is present	495 (31.6%)	341 (34.4%)	154 (26.8%)
Syndromic or genetic condition	104 (6.6%)	88 (8.9%)	16 (2.8%)

**Table 2:** Retrospectively perceived condition before and after COVID-19 infection. Percentages refer to respondents who reported that the patient/child/adolescent had experienced a COVID-19 infection.

**Parents Assess the Impact of the Child’s COVID-19 Infection on Physical Resilience, Psychological State, Social Environment and General Health**							
		**Total** **N = 737**	**Male** **n = 394**	**Female** **n = 343**	**Simple CHD** **n = 186**	**Moderate CHD** **n = 254**	**Complex CHD** **n = 297**
Physical resilience (sport, leisure time, everyday life) is now… male vs. female: *p* = 0.018	worse	72 (9.8%)	29 (7.4%)	43 (12.5%)	19 (10.2%)	24 (9.4%)	29 (9.8%)
	unchanged	636 (86.3%)	345 (87.6%)	291 (84.8%)	161 (86.6%)	221 (87.0%)	254 (85.5%)
	better	29 (3.9%)	20 (5.1%)	9 (2.6%)	6 (3.2%)	9 (3.5%)	14 (4.7%)
Psychological state (feelings, fears, worries, desires) is now…	worse	139 (18.9%)	69 (17.5%)	70 (20.4%)	31 (16.7%)	53 (20.9%)	55 (18.5%)
	unchanged	566 (76.8%)	306 (77.7%)	260 (75.8%)	144 (77.4%)	192 (75.6%)	230 (77.4%)
	better	32 (4.3%)	19 (4.8%)	13 (3.8%)	11 (5.9%)	9 (3.5%)	12 (4.0%)
Social environment (acquaintances, friends, family) is now…	worse	107 (14.5%)	53 (13.5%)	54 (15.7%)	27 (14.5%)	39 (15.4%)	41 (13.8%)
	unchanged	586 (79.5%)	313 (79.4%)	273 (79.6%)	148 (79.6%)	198 (78.0%)	240 (80.8%)
	better	44 (6.0%)	28 (7.1%)	16 (4.7%)	11 (5.9%)	17 (6.7%)	16 (5.4%)
General health (physical and mental well-being) is now…	worse	132 (17.9%)	64 (16.2%)	68 (19.8%)	25 (13.4%)	55 (21.7%)	52 (17.5%)
	unchanged	562 (76.3%)	302 (76.6%)	260 (75.8%)	150 (80.6%)	186 (73.2%)	226 (76.1%)
	better	43 (5.8%)	28 (7.1%)	15 (4.4%)	11 (5.9%)	13 (5.1%)	19 (6.4%)
**Patients assess the impact of their COVID-19 infection on physical resilience, psychological state, social environment and general health**							
		total N = 417	male n = 153	female n = 264	simple CHD n = 166	moderate CHD n = 118	complex CHD n = 133
Physical resilience (sport, leisure time, everyday life) is now… male vs. female: *p* = 0.0004	worse	69 (16.5%)	12 (7.8%)	57 (21.6%)	37 (22.3%)	15 (12.7%)	17 (12.8%)
	unchanged	311 (74.6%)	122 (79.7%)	189 (71.6%)	112 (67.5%)	93 (78.8%)	106 (79.7%)
	better	37 (8.9%)	19 (12.4%)	18 (6.8%)	17 (10.2%)	10 (8.5%)	10 (7.5%)
Psychological state (feelings, fears, worries, desires) is now… simple vs. moderate vs. complex CHD: *p* = 0.031	worse	89 (21.3%)	24 (15.7%)	65 (24.6%)	45 (27.1%)	23 (19.5%)	21 (15.8%)
	unchanged	297 (71.2%)	118 (77.1%)	179 (67.8%)	104 (62.7%)	89 (75.4%)	104 (78.2%)
	better	31 (7.4%)	11 (7.2%)	20 (7.6%)	17 (10.2%)	6 (5.1%)	8 (6.0%)
Social environment (acquaintances, friends, family) is now…	worse	59 (14.1%)	17 (11.1%)	42 (15.9%)	30 (18.1%)	16 (13.6%)	13 (9.8%)
	unchanged	277 (66.4%)	102 (66.7%)	175 (66.3%)	105 (63.3%)	81 (68.6%)	91 (68.4%)
	better	81 (19.4%)	34 (22.2%)	47 (17.8%)	31 (18.7%)	21 (17.8%)	29 (21.8%)
General health (physical and mental well-being) is now… male vs. female: *p* = 0.0001; simple vs. moderate vs. complex CHD: *p* = 0.007	worse	83 (19.9%)	15 (9.8%)	68 (25.8%)	45 (27.1%)	14 (11.9%)	24 (18.0%)
	unchanged	288 (68.6%)	114 (74.5%)	172 (65.2%)	98 (59.0%)	93 (78.8%)	95 (71.4%)
	better	48 (11.5%)	24 (15.7%)	24 (9.1%)	23 (13.9%)	11 (9.3%)	14 (10.5%)

**Table 3:** Reasons for psychological, psychotherapeutic, or psychiatric treatment (PST); multiple answers were possible.

**Reasons for PST of the CHD Patient According to the Parents’ Statements**						
	**Total** **N = 236**	**Male** **n = 135**	**Female** **n = 101**	**Simple CHD** **n = 50**	**Moderate CHD** **n = 70**	**Complex CHD** **n = 116**
Problems at home/with the family	47 (19.9%)	30 (22.2%)	17 (16.8%)	13 (26.0%)	17 (24.3%)	17 (14.7%)
Problems at school/training/university	78 (33.1%)	51 (37.8%)	27 (26.7%)	15 (30.0%)	22 (31.4%)	41 (35.3%)
Problems with other students/acquaintances/friends	51 (21.6%)	29 (21.5%)	22 (21.8%)	13 (26.0%)	14 (20.0%)	24 (20.7%)
Because of a mental illness	55 (23.3%)	30 (22.2%)	25 (24.8%)	13 (26.0%)	16 (22.9%)	26 (22.4%)
Because of the congenital heart defect simple vs. moderate vs. complex CHD: *p* = 0.0004	61 (25.8%)	41 (30.4%)	20 (19.8%)	4 (8.0%)	15 (21.4%)	42 (36.2%)
For other reasons	84 (35.6%)	44 (32.6%)	40 (39.6%)	21 (42.0%)	22 (31.4%)	41 (35.3%)
**Reasons for PST of the CHD patient according to the patients’ statements**						
	total N = 149	male n = 55	female n = 94	simple CHD n = 61	moderate CHD n = 34	complex CHD n = 54
Problems at home/with the family male vs. female: *p* = 0.010; simple vs. moderate vs. complex CHD: *p* = 0.028	43 (28.9%)	9 (16.4%)	34 (36.2%)	24 (39.3%)	10 (29.4%)	9 (16.7%)
Problems at school/training/university	61 (40.9%)	22 (40.0%)	39 (41.5%)	27 (44.3%)	12 (35.3%)	22 (40.7%)
Problems with other students/acquaintances/friends	34 (22.8%)	13 (23.6%)	21 (22.3%)	13 (21.3%)	11 (32.4%)	10 (18.5%)
Because of a mental illness	45 (30.2%)	13 (23.6%)	32 (34.0%)	23 (37.7%)	10 (29.4%)	12 (22.2%)
Because of the congenital heart defect male vs. female: *p* = 0.005; simple vs. moderate vs. complex CHD: *p* = 0.004	22 (14.8%)	14 (25.5%)	8 (8.5%)	2 (3.3%)	8 (23.5%)	12 (22.2%)
For other reasons	46 (30.9%)	17 (30.9%)	29 (30.9%)	17 (27.9%)	9 (26.5%)	20 (37.0%)

**Table 4:** Patient- and parent-rated support, helpfulness, and perceived long-term improvement associated with psychological, psychotherapeutic, or psychiatric treatment (PST).

**Parent-Rated Support, Helpfulness, and Perceived Long-Term Improvement Associated with PST of the CHD Patient**							
		**Total** **N = 236**	**Male** **n = 135**	**Female** **n = 101**	**Simple CHD** **n = 50**	**Moderate CHD** **n = 70**	**Complex CHD** **n = 116**
Support level *Patient felt well supported/advised during PST* male vs. female: *p* = 0.007	low	18 (7.6%)	15 (11.1%)	3 (3.0%)	3 (6.0%)	5 (7.1%)	10 (8.6%)
	moderate	86 (36.4%)	55 (40.7%)	31 (30.7%)	14 (28.0%)	25 (35.7%)	47 (40.5%)
	high	132 (55.9%)	65 (48.1%)	67 (66.3%)	33 (66.0%)	40 (57.1%)	59 (50.9%)
Help level *How well did the PST help the patient*	low	38 (16.1%)	27 (20.0%)	11 (10.9%)	5 (10.0%)	10 (14.3%)	23 (19.8%)
	moderate	86 (36.4%)	51 (37.8%)	35 (34.7%)	14 (28.0%)	26 (37.1%)	46 (39.7%)
	high	112 (47.5%)	57 (42.2%)	55 (54.5%)	31 (62.0%)	34 (48.6%)	47 (40.5%)
Long-term improvements *Has PST led to longer-term improvement* male vs. female: *p* = 0.005; simple vs. moderate vs. complex CHD: *p* = 0.037	low	51 (21.6%)	39 (28.9%)	12 (11.9%)	5 (10.0%)	12 (17.1%)	34 (29.3%)
	moderate	89 (37.7%)	49 (36.3%)	40 (39.6%)	19 (38.0%)	27 (38.6%)	43 (37.1%)
	high	96 (40.7%)	47 (34.8%)	49 (48.5%)	26 (52.0%)	31 (44.3%)	39 (33.6%)
**Patient-rated support, helpfulness, and perceived long-term improvement associated with PST**							
		total N = 149	male n = 55	female n = 94	simple CHD n = 61	moderate CHD n = 34	complex CHD n = 54
Support level *Patient felt well supported/advised during PST*	low	17 (11.4%)	5 (9.1%)	12 (12.8%)	9 (14.8%)	1 (2.9%)	7 (13.0%)
	moderate	48 (32.2%)	14 (25.5%)	34 (36.2%)	22 (36.1%)	14 (41.2%)	12 (22.2%)
	high	84 (56.4%)	36 (65.5%)	48 (51.1%)	30 (49.2%)	19 (55.9%)	35 (64.8%)
Help level *How well did the PST help the patient*	low	27 (18.1%)	9 (16.4%)	18 (19.1%)	15 (24.6%)	4 (11.8%)	8 (14.8%)
	moderate	50 (33.6%)	18 (32.7%)	32 (34.0%)	18 (29.5%)	12 (35.3%)	20 (37.0%)
	high	72 (48.3%)	28 (50.9%)	44 (46.8%)	28 (45.9%)	18 (52.9%)	26 (48.1%)
Long-term improvements *Has PST led to longer-term improvement* simple vs. moderate vs. complex CHD: *p* = 0.005	low	34 (22.8%)	8 (14.5%)	26 (27.7%)	21 (24.4%)	5 (14.7%)	8 (14.8%)
	moderate	56 (37.6%)	20 (36.4%)	36 (38.3%)	14 (23.0%)	13 (38.2%)	29 (53.7%)
	high	59 (39.6%)	27 (49.1%)	32 (34.0%)	26 (42.6%)	16 (47.1%)	17 (31.5%)

**Table 5:** Exploratory multivariable logistic regression analyses of selected key outcomes.

Outcome	Respondent Group	Predictor	OR	95% CI	*p*-Value
School-related stress	Patient self-reports ≥ 11 years, N = 503	Age, per year	1.19	1.07–1.32	0.002
		Female sex	1.71	1.17–2.52	0.006
		Moderate CHD vs. simple CHD	0.78	0.50–1.24	0.296
		Complex CHD vs. simple CHD	0.88	0.57–1.38	0.586
		Syndromic/genetic condition	0.87	0.29–2.66	0.813
Less good/poor self-rated health	Patient self-reports ≥ 11 years, N = 503	Age, per year	1.33	1.10–1.61	0.003
		Female sex	1.79	0.98–3.30	0.060
		Moderate CHD vs. simple CHD	1.79	0.87–3.68	0.112
		Complex CHD vs. simple CHD	3.16	1.62–6.14	0.001
		Syndromic/genetic condition	0.34	0.04–2.77	0.314
Less good/poor health	Parent reports, N = 992	Age, per year	1.07	1.00–1.13	0.037
		Female sex	1.16	0.76–1.75	0.494
		Moderate CHD vs. simple CHD	0.79	0.42–1.47	0.453
		Complex CHD vs. simple CHD	1.96	1.16–3.32	0.013
		Syndromic/genetic condition	1.27	0.63–2.55	0.511
High COVID-19-related burden	Patient self-reports ≥ 11 years, N = 503	Age, per year	1.12	0.98–1.27	0.093
		Female sex	2.49	1.53–4.05	<0.001
		Moderate CHD vs. simple CHD	0.70	0.39–1.23	0.213
		Complex CHD vs. simple CHD	1.45	0.87–2.41	0.151
		Syndromic/genetic condition	0.80	0.21–3.10	0.749
Any self-reported mental illness	Patient self-reports, N = 575	Age, per year	1.09	1.00–1.19	0.065
		Female sex	1.31	0.82–2.10	0.263
		Moderate CHD vs. simple CHD	0.74	0.41–1.33	0.309
		Complex CHD vs. simple CHD	1.31	0.78–2.22	0.312
Previous/current PST use	Patient self-reports, N = 575	Age, per year	1.10	1.02–1.19	0.014
		Female sex	1.14	0.77–1.70	0.514
		Moderate CHD vs. simple CHD	0.87	0.53–1.44	0.590
		Complex CHD vs. simple CHD	1.39	0.87–2.20	0.165
		Syndromic/genetic condition	0.64	0.18–2.36	0.506
Previous/current PST use	Parent reports, N = 992	Age, per year	1.12	1.07–1.17	<0.001
		Female sex	0.91	0.67–1.23	0.520
		Moderate CHD vs. simple CHD	1.35	0.88–2.06	0.167
		Complex CHD vs. simple CHD	2.10	1.41–3.11	<0.001
		Syndromic/genetic condition	0.50	0.27–0.92	0.027
Moderate/high perceived long-term improvement after PST	Patient self-reports with previous/current PST, N = 149	Age, per year	0.86	0.69–1.08	0.186
		Female sex	0.74	0.28–1.94	0.537
		Moderate CHD vs. simple CHD	2.28	0.73–7.12	0.156
		Complex CHD vs. simple CHD	1.96	0.70–5.49	0.198
Moderate/high perceived long-term improvement after PST	Parent reports with previous/current PST, N = 236	Age, per year	1.08	0.98–1.19	0.131
		Female sex	2.73	1.32–5.66	0.007
		Moderate CHD vs. simple CHD	0.77	0.24–2.43	0.650
		Complex CHD vs. simple CHD	0.37	0.13–1.04	0.060
		Syndromic/genetic condition	0.51	0.14–1.85	0.304

ORs are from exploratory multivariable logistic regression models. Models were fitted separately for patient self-reports and parent reports where applicable. Simple CHD, male sex, and absence of syndromic/genetic condition served as reference categories. School-related stress was coded as somewhat/very stressful versus moderate/not very/not at all stressful. Less good/poor health was coded as less good/poor versus good/very good/excellent. High COVID-19-related burden was coded as quite/extremely stressful versus somewhat/not at all/hardly stressful. Any self-reported mental illness and previous/current PST use were coded as yes versus no. Moderate/high perceived long-term improvement after PST was coded as moderate/high versus low perceived long-term improvement. The model for a self-reported mental illness was adjusted for age, sex, and CHD severity only because inclusion of syndromic/genetic conditions resulted in quasi-complete separation and model non-convergence. The patient self-report model for perceived long-term improvement after PST was also adjusted for age, sex, and CHD severity only because inclusion of syndromic/genetic conditions resulted in model non-convergence. CI = confidence interval; CHD = congenital heart defect; OR = odds ratio; PST = psychological, psychotherapeutic, or psychiatric treatment.

## Data Availability

Data cannot be shared for data protection reasons.
